# A Pioneer in Revolutionizing Physiotherapy in India: Dr. M. G. Mokashi's Journey of Compassion, Innovation, and Dedication

**DOI:** 10.7759/cureus.67560

**Published:** 2024-08-23

**Authors:** Gurjeet Kaur, Moh'd Irshad Qureshi, Raghumahanti Raghuveer, Pratik Phansopkar, H V Sharath

**Affiliations:** 1 Department of Physiotherapy, Center for Advanced Physiotherapy Education and Research, Ravi Nair Physiotherapy College, Datta Meghe Institute of Higher Education and Research (Deemed to be University), Wardha, IND; 2 Department of Neuro-Physiotherapy, Center for Advanced Physiotherapy Education and Research, Ravi Nair Physiotherapy College, Datta Meghe Institute of Higher Education and Research (Deemed to be University), Wardha, IND; 3 Department of Musculoskeletal Physiotherapy, Center for Advanced Physiotherapy Education and Research, Ravi Nair Physiotherapy College, Datta Meghe Institute of Higher Education and Research (Deemed to be University), Wardha, IND; 4 Department of Pediatric Physiotherapy, Center for Advanced Physiotherapy Education and Research, Ravi Nair Physiotherapy College, Datta Meghe Institute of Higher Education and Research (Deemed to be University), Wardha, IND

**Keywords:** neurophysiotherapy, rehabilitation, m. g. mokashi, historical vignette, physiotherapist, therapeutic biofeedback, biofeedback therapy

## Abstract

Dr. Madhav Gajanan Mokashi is a remarkable individual in physiotherapy, and he is celebrated for his exceptional blend of educational and clinical excellence. His innovative research and influential roles in prominent conferences have shaped the physiotherapy landscape in India. Beyond his professional achievements, Dr. Mokashi has touched countless lives with his unique approach, integrating spiritual wisdom with practical expertise to foster an atmosphere of empathy and respect. His journey, marked by significant health challenges and an amputation, stands as a testament to his unwavering dedication, resilience, and sense of humor. Dr. Mokashi’s legacy extends beyond his professional milestones; it resonates deeply in the hearts of those he has mentored and inspired. This review honours his enduring impact and the profound inspiration he offers to the next generation of healthcare professionals.

## Introduction and background

Dr. Madhav Gajanan Mokashi was a revered figure in Indian physiotherapy, affectionately known as MGM among his colleagues. He resided in Mumbai with his wife, Dr. Umalini. He continued to inspire others with his profound insights and dedication to education, exemplifying his commitment to the profession and reflecting his passion for physiotherapy. Even at 78, he believed that learning is a lifelong journey [1]. Throughout his career, Dr. Mokashi emphasized the importance of approaching problems with an open mind, free from prejudice. He often articulated, “If your mind is filled with biased thoughts, it is like a full pot which will not be able to hold any more.” This philosophy profoundly influenced his students and colleagues in patient care, interpersonal relationships, and day-to-day interactions [2]. Dr. Mokashi's unwavering dedication to physiotherapy was evident in his relentless efforts at the Central Government level. Even while battling health challenges, he wished to help the profession until his last breath. His resilience and humor were remarkable, even as he shared his experiences regarding amputation [2]. This resilience is shown in the many documented challenges in beginner physiotherapy education in India, where theory and practice have been carefully examined [3].

**Figure 1:**
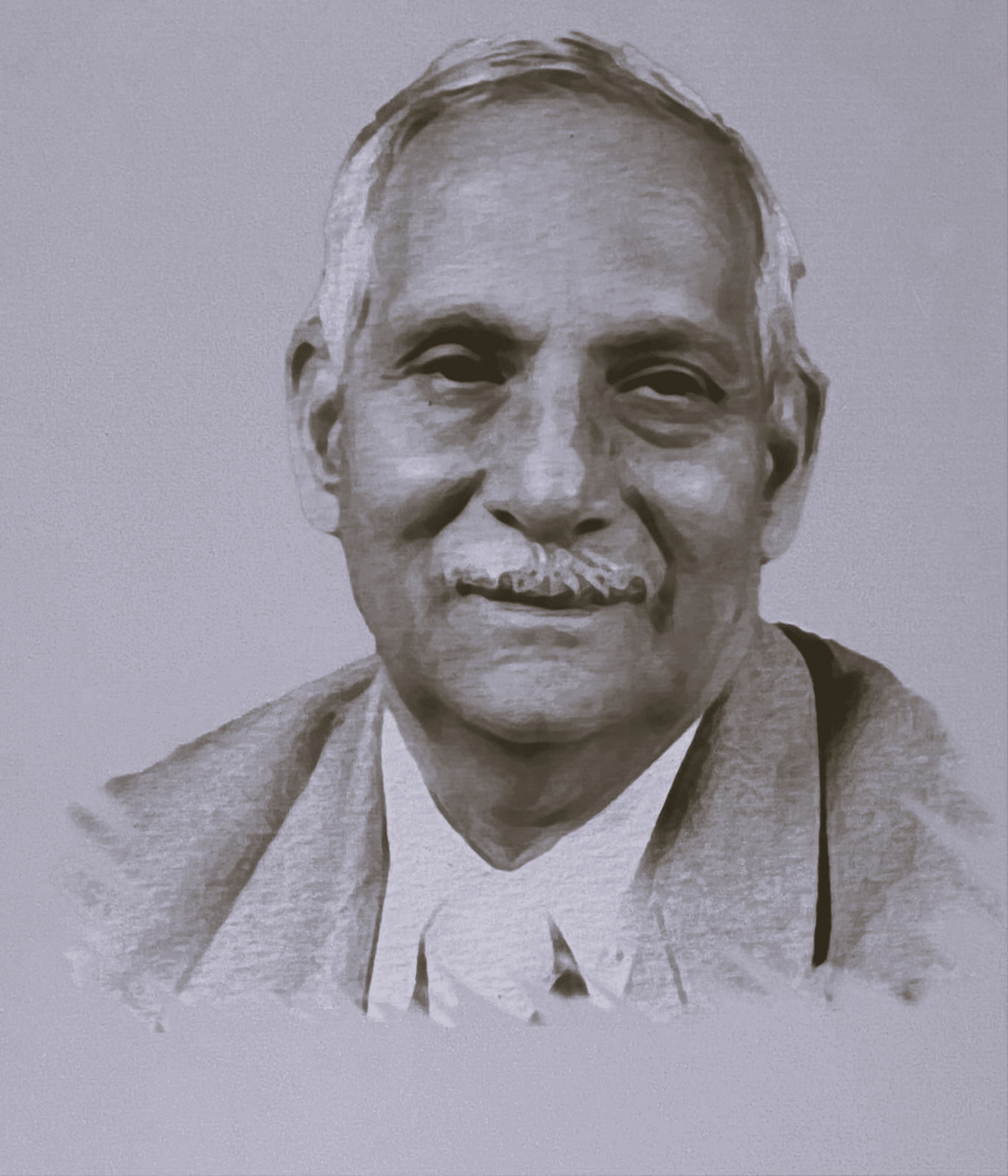
Dr. Madhav Gajanan Mokashi Image courtesy of Holistic Approach to Neurophysiotherapy: Evidence and Experiences [1]

Prof. C. K. Senthil Kumar, PhD, Dean of the Bethel Medical Mission Group, Bangalore, in his interview with PHYSILIFE, described Dr. Mokashi as the "Godfather for the profession." He recounted how Dr. Mokashi selflessly supported students with guidance and financial assistance as a father figure. He praised Dr. Mokashi's principles and commitment to professional development despite personal health struggles. He started his Indian Association of Physiotherapists (IAP) career under the guidance of Guru Late Dr. M. G. Mokashi and Dr. Subodh Desai [4,5]. Figure 1 shows the image of Prof. Late M. G. Mokashi. Biography paints a vivid picture of the journey of physiotherapy in India, seen through the lens of his personal battles, experiences, and success. His story is more than just a recount of his life. It is a heartfelt source of inspiration and guidance for those who dream of making a difference in physiotherapy and healthcare.

## Review

Early life and education

Dr. Mokashi belonged to a humble family where hard work and the pursuit of knowledge were highly valued. His journey in physiotherapy and neurology began with a Bachelor of Science degree in 1958, followed by a Diploma in Physiotherapy from King Edward Memorial Hospital in Mumbai. By 1960, he had also completed a PG Certificate in Rehabilitation from the All India Institute of Physical Medicine and Rehabilitation (AIIPMR). In 1986, he completed his PhD in the Psychophysical and Philosophical Aspects of Patanjali Yoga from the University of Mumbai. In 2000, he expanded his academic horizons by receiving a Doctor of Science (Alternate Medicine) for his innovative work on "Therapeutic Biofeedback and Therapeutic Yoga."

Dr. Mokashi was trained in “Clinical Prosthetics” by James Foort at the University of Saskatoon. He also studied “Biomechanics for Bioengineering Appliances” at the Crippled Children's Centre in Toronto under C. A. McLaurin. He became part of Dr. Sant's selected team of engineers and technicians, including T. P. Mirajkar, Joseph T. Carvalho, and S. T. Nagale, contributing to the evolution of orthotic technology in India, which historically transitioned from blacksmiths and carpenters to trained engineers and medical professionals [6].

In 1980, Dr. Mokashi was the first physiotherapist to receive a three-year ICMR project as the Principal Investigator for "Therapeutic Biofeedback." He represented India by presenting the "Plastic Lower Limb Orthoses" project report at the 1989 WHO Intercountry Workshop on Orthopaedic Technologies in Dakar, Senegal. Research and Dr. M. G. Mokashi were inseparable throughout his career [2].

Professional beginnings

Dr. M. G. Mokashi started his professional career at AIIPMR in Mumbai under the mentorship of the Institute's founder, the late Dr. M. V. Sant, whose guidance significantly influenced his approach to patient care, research, and education during a time when physiotherapy was still gaining recognition in India. From 1961 to 1994, Dr. Mokashi was the chief of physiotherapy and a postgraduate teacher at AIIPMR. He later served as the Principal of Laxmi Memorial College of Physiotherapy in Mangalore, HOD, and later Professor Emeritus-Director at Jawaharlal Nehru Medical College in Belgaum, Karnataka, in 2005, and adjunct professor guiding PhD programs at Dr. D. Y. Patil University in Pune [1,2,7].

Dr. Mokashi was a Professor Emeritus at Padmashree Dr. D. Y. Patil College of Physiotherapy, where he participated in scientific committee and Reward and Recognition meetings [8]. At KLE Academy of Higher Education & Research in Belagavi, he, as a guest faculty member, delivered a lecture on “Continuing on the Philosophy of Research” on October 12, 2012. He was also involved in various committees for the Indian Council for Medical Research (ICMR), the Department of Science and Technology, and public service commissions in Maharashtra and Kerala [9].

Commitment to learning and teaching

Dr. M. G. Mokashi believed in "learning by doing," emphasizing practical experience over traditional teaching. He encouraged students and practitioners to engage with the community and learn from each patient's unique condition, which led to his active participation in rural health camps across Maharashtra. He focussed on community-based rehabilitation and patient-centric care [2].

His dedication to education was evident in developing undergraduate and postgraduate physiotherapy program curricula. In 2022, he guided PhD students at Jawaharlal Nehru Medical College in Belgaum, mentoring research on topics such as sensory threshold values to short- and long-pulse duration in case of trigeminal, radial, median, and ulnar nerves in normal subjects and determination of sensory strength-duration curve by Patil Chandragouda B.; a study of autonomic nervous dysfunction by Valsalva maneuver response to sustained hand grip in middle-aged subject's having hypertension and diabetes mellitus by Ganesh B. R.; comparative efficacy of neural mobilization and McKenzie manipulation in cervical radiculopathy by Kumar Sanjiv [10]; studies on stress in physiotherapy students by self-reported questionnaires, clinical parameters by Tushar Jaikrishna Palekar; and role of yoga asanas in testing neural tissue tension and mobilization of peripheral nerves by Gaurang Dinesh Baxi and many more [1].

Pioneering contributions and research

He has also served as an expert to government organizations, universities, and nongovernmental organizations, and he has been a Visiting Professor of biomedical engineering. The first IAP journal, published in 1958 under the guidance of Mr. A. V. Majumdar (first Editor-in-Charge), was later edited by Mr. Manik Shahani and Dr. Mokashi. From 1965 to 2006, Dr. Mokashi significantly contributed to physiotherapy, serving as chairman and member of the Board of Academics for the IAP. He is on the Editorial Board of the Indian Journal of Physiotherapy and Occupational Therapy, the International Journal of Health Sciences and Research, and the Journal of KLE University. He helped found the first IAP journal in 1958, serving as its editor from 1970 to 1982 and again in 1999 and 2004. He also served as a convener for several IAP National Committees, including those on Education, Scientific Programmes, the Council Act, Infrastructure Development and Research, and Pay Structure [1,2,11].

In 1974, he became the first physiotherapist to present original research at international forums, including the World Confederation for Physical Therapy (WCPT), with his groundbreaking paper on "Yoga and Physiotherapy." His dedication to education and research was honored in 1983 when he was awarded the Fellow of IAP for his significant contributions. In 1980, he was awarded a three-year ICMR Project as the Principal Investigator on "Therapeutic Biofeedback." He was trained by pioneering foreign researchers, gained vast research experience, and published over 120 papers, earning recognition at national and international conferences.

In 2000, he earned a Doctor of Science degree in Alternate Medicine and received a Colombo Plan Fellowship to study kinesiology and clinical electromyography at Queen’s University in Canada. Meanwhile, the American Physical Therapy Association accepted his original research paper, which was presented in San Francisco. He authored his first book (452 pages), "Holistic Approach to Neuro-physiotherapy: Experiences and Evidences," which compiles practical insights and scientific evidence for neurophysiology and rehabilitation professionals. It encompasses his personal experiences with various conditions, supported by scientific evidence, and includes a chapter on his collaboration with Dr. Sant [1,2]. This comprehensive book showcases Dr. Mokashi's dedication and expertise in the field [12].

With a notable presence in academic literature, a search M.G. Mokashi author:M.G author:Mokashi reveals 14 articles on Google Scholar and four published articles in the "Fizjoterapeuta" journal, covering topics like physiotherapy in Parkinson's disease, interdisciplinary guidelines for lumbar pain, the past and present possibilities in cerebral palsy, neurological patients, and the role of neurophysiotherapists, demonstrating his ongoing impact on the field [13].

Leadership and advocacy

Dr. M. G. Mokashi was a passionate advocate for the growth of physiotherapy in India, extending his influence beyond clinical and academic realms. He served as the President of the IAP three times (1968-1969, 1978-1980, and 1994-1996). He represented IAP at the WCPT Congress in Montreal (1974) and Washington, DC (1995). He participated in the WHO Workshop on Orthopaedic Technologies in Dakar, Senegal (1989) and was a Patron of the IAP Annual Conference in Jaipur (2004) [1,2].

Since 2011, Dr. Mokashi has been the Chief Mentor of PHYSIOTIMES [14], a bimonthly physiotherapy magazine from Ahmedabad. He contributed to chapters on Yoga in Neurological Disorders to books published by Punjabi University, the National Diabetes Foundation in Belgaum, and Father Muller College in Mangalore. His leadership has shaped policies and educational standards in physiotherapy, aiming to establish an independent physiotherapy council [1,2].

In PHYSIOTIMES, Dr. Mokashi wrote about India’s role in WCPT over 40 years, highlighting new pain management and rehabilitation technologies, such as high-intensity laser therapy, inversion therapy for spinal decompression, and the EndoRush rehabilitation app. The article also featured an interview with ergonomics pioneer Prof. P. N. Saha and included insights on cardiovascular ergonomics. These contributions honored Dr. Mokashi's legacy and his encouragement for authors to incorporate ergonomics into their work [15].

Awards and conferences named in his honor

Dr. M. G. Mokashi's legacy is honored through the Dr. M.G.M. International Physiocon Conference and the Dr. M. G. Mokashi Best Physiotherapist/Physiotherapy Teacher Award. Dr. Sanjiv Kumar received this award at the National Conference Physiocon 2014 in Bangalore on November 11, and he, along with Dr. Jeba Chitra, was certified with a best physiotherapist and teacher award in December 2014 at the HOSMAT conference in Bangalore [16]. Dr. Ali Irani was the first Dr. M. G. Mokashi Professional Excellence Award recipient at Dr. MGM International Physiocon 2016 [17]. Dr. Jeba Chitra was honored again on March 4, 2018, at the NIMHANS conference. The Dr. M.G.M. International Physiocon, dedicated to promoting collaboration among physiotherapists, was held in 2020 [18].

On January 19 and 20, 2016, T. Poovishnu Devi received the award at a conference in Udaipur, Rajasthan. The award was also given to Dr. G. Varadharajuluz, S. Anandh, and Vijay Selvan at a subsequent conference in Mapusa, Goa, from January 20 to 22. On February 1, 2020, Dr. Poovishnu Devi received the Dr. M. G. Mokashi Best Interdisciplinary Innovation Award at a conference at Sapthagiri Medical College, Bangalore [19,20].

The 10th Dr. MGM International Physiotherapy Conference held at North East Christian University in Dimapur on January 23, with the Governor of Nagaland, La Ganesan, emphasized the conference's significance in honoring the late Mahadev G. Mokashi and highlighted the need for students to stay updated with advancements in physiotherapy, under the theme, "Changing Trends: Challenging the Future."

Legacy and personal values

The legacy of physiotherapy in India, shaped by pioneers like the Late Prof. M. G. Mokashi and Dr. D. H. Dastoor, is evolving under President Dr. Sanjiv K. Jha. This new era emphasizes advancing the profession, tackling societal health issues, securing funding for research, and enhancing healthcare access through reform.

Dr. Mokashi was not only professionally accomplished but also deeply spiritual and compassionate. A scholar of Sanskrit and Marathi literature, he translated the Bhagavad Gita into Marathi and shared his insights through poetry. Known for his unique intuition and yogic beliefs, i.e., influenced by yoga and holistic wellness, he has enriched the lives of his students and peers with his wisdom and kindness. Madam Mokashi supported all her students with love and warmth, treating them like family. Even though Dr. Mokashi faced enormous health struggles, including losing a limb to sarcoma, he remained an inspiring figure. His unwavering dedication to his students and the field of physiotherapy was truly remarkable. Through it all, his resilience, humor, and commitment shone brightly, touching everyone around him until the end [2].

## Conclusions

Dr. M. G. Mokashi’s legacy in physiotherapy is a witness to his unwavering commitment to education, holistic healing, and compassionate care. His groundbreaking work reflects his exceptional professionalism and his deep impact on students and colleagues alike. Dr. Mokashi created a nurturing environment filled with respect and open-mindedness by blending spiritual insight with practical expertise. Even in the face of severe health challenges, including an amputation, his resilience shone through, inspiring many with his kindness and humour. His dedication ensures that his influence will continue to shape the future of physiotherapy, leaving behind a lasting mark of integrity, compassion, and excellence in the field.
